# g:Profiler—interoperable web service for functional enrichment analysis and gene identifier mapping (2023 update)

**DOI:** 10.1093/nar/gkad347

**Published:** 2023-05-05

**Authors:** Liis Kolberg, Uku Raudvere, Ivan Kuzmin, Priit Adler, Jaak Vilo, Hedi Peterson

**Affiliations:** Institute of Computer Science, University of Tartu, Narva mnt 18, 51009 Tartu, Estonia; Institute of Computer Science, University of Tartu, Narva mnt 18, 51009 Tartu, Estonia; Institute of Computer Science, University of Tartu, Narva mnt 18, 51009 Tartu, Estonia; Institute of Computer Science, University of Tartu, Narva mnt 18, 51009 Tartu, Estonia; Institute of Computer Science, University of Tartu, Narva mnt 18, 51009 Tartu, Estonia; Software Technology and Applications Competence Center, Narva mnt 20, 51009 Tartu, Estonia; Institute of Computer Science, University of Tartu, Narva mnt 18, 51009 Tartu, Estonia

## Abstract

g:Profiler is a reliable and up-to-date functional enrichment analysis tool that supports various evidence types, identifier types and organisms. The toolset integrates many databases, including Gene Ontology, KEGG and TRANSFAC, to provide a comprehensive and in-depth analysis of gene lists. It also provides interactive and intuitive user interfaces and supports ordered queries and custom statistical backgrounds, among other settings. g:Profiler provides multiple programmatic interfaces to access its functionality. These can be easily integrated into custom workflows and external tools, making them valuable resources for researchers who want to develop their own solutions. g:Profiler has been available since 2007 and is used to analyse millions of queries. Research reproducibility and transparency are achieved by maintaining working versions of all past database releases since 2015. g:Profiler supports 849 species, including vertebrates, plants, fungi, insects and parasites, and can analyse any organism through user-uploaded custom annotation files. In this update article, we introduce a novel filtering method highlighting Gene Ontology driver terms, accompanied by new graph visualizations providing a broader context for significant Gene Ontology terms. As a leading enrichment analysis and gene list interoperability service, g:Profiler offers a valuable resource for genetics, biology and medical researchers. It is freely accessible at https://biit.cs.ut.ee/gprofiler.

## Graphical Abstract

**Graphical Abstract:**
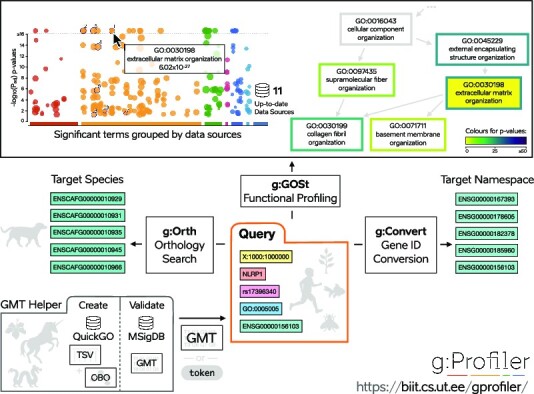
g:Profiler is a versatile web service offering functional profiling, gene ID conversion, and orthology search capabilities. Compatible with all identifier types recognized by Ensembl, it produces detailed tables and generates interactive Manhattan plots and GO context graphs to capture essence of the results. Integration of external data is facilitated by the GMT Helper application.

## INTRODUCTION

Functional enrichment analysis plays a crucial role in contemporary biological research by aiding in the identification of relevant molecular mechanisms, biological processes and pathways related to the research question at hand. This step is vital in contextualizing gene lists and refining the resulting conclusions. To accomplish this, valuable datasets are collected and curated to form dedicated knowledge bases such as Ensembl (1), Reactome (2) and CORUM (3), and structured vocabularies such as the Human Phenotype Ontology (4) and Gene Ontology (GO) (5). Enrichment analysis tools utilize these datasets to provide valuable insights to the scientific community.

Since the inception of GO, many enrichment analysis tools have been developed to support biological research (6–10). However, while several of these tools have been developed further and continue to provide regular data updates, many others have not and may provide outdated results (11). As a leading resource for functional enrichment analysis, g:Profiler has emerged as a user-friendly and efficient tool that stands out from the competition. Notably, g:Profiler provides regular data updates, ensuring its users can access the latest datasets to support their analyses (12). It also allows users to provide custom background for statistical analysis, always returns only corrected *P*-values, and provides data, code and parameter versions in an open manner. For a detailed comparison of g:Profiler with other enrichment analysis tools, please refer to Supplementary Table S1. We understand the significance of reproducibility in research and have made it a priority to provide archived versions of previous releases, allowing for easy reproducibility of analysis results.

Our user interface has been designed with a focus on intuitive use, helping researchers from a range of backgrounds to utilize the tool effectively. At g:Profiler, we place great importance on the input and feedback of our users and are fully committed to providing swift responses to their requests and suggestions. Our dedication to this approach has resulted in the successful implementation of many innovative features, such as the Manhattan plot, custom Gene Matrix Transposed (GMT) files and programmatic access points via our Python and R clients. In this update article, we present three novel features that enhance the g:Profiler user experience. These features include a two-stage algorithm for filtering GO enrichment results, a graph view that visualizes significant biological functions, hereinafter called terms, and their relationships within the ontology, and the GMT Helper toolkit, which allows users to customize annotations for less studied species. These features empower biologists to gain a comprehensive summary of their data and better understand their results’ biological context.

By incorporating these user-friendly and cutting-edge features, g:Profiler continues to position itself as a leading resource for gene set enrichment analysis in the biological research community. These innovations have proven immensely popular, with over 24 million queries processed throughout the year of 2022 alone, which is >10 times the number of queries processed by one of the most widely known tool, DAVID, for example (10). The ease of use and up-to-date data have led GO initiative itself to select g:Profiler as one of their top choices, placing it among the five recommended enrichment analysis tools featured on their website.

In summary, g:Profiler is an essential tool for biologists seeking to identify and understand the molecular mechanisms and biological processes underlying their research. With its powerful features, intuitive interface and focus on user needs, g:Profiler stands out as a leading web service for gene set enrichment analysis. It is also recognized as an ELIXIR Recommended Interoperable Service that provides translation between tens of gene identifiers and allows fast ortholog mapping queries.

## THE CORE OF g:PROFILER

g:Profiler is a collection of widely used tools that have become essential to standard pipelines in computational biology research focused on genes and proteins. One of the key tools available is g:GOSt, widely used for performing functional enrichment analyses on gene lists. g:Convert is used for mapping gene/protein identifiers across different namespaces, while g:Orth enables the mapping of orthologous genes across different species. In addition, g:SNPense is a valuable tool for mapping human single-nucleotide polymorphism identifiers to their corresponding genes and providing their predicted variant effects.

g:Profiler relies on the comprehensive datasets provided by Ensembl (1) and Ensembl Genomes (13) via the Biomart database files. These datasets contain high-quality data for almost 1000 species, including hundreds of namespaces for the most widely studied organisms, ortholog mappings between different organisms, and annotations for GO (5,14) and Reactome (2), all quarterly updated. Using Ensembl as our primary data source allows for the seamless combination of namespaces in the input forms of our tool. This gives users the freedom to use the gene identifier or naming standard they have at hand without the need to convert all IDs to a certain namespace.

In addition to Ensembl, g:Profiler includes other reliable and well-maintained datasets, such as those offered by WormBase ParaSite (15,16) and other specialized databases. To keep pace with the ever-expanding and evolving data sources, g:Profiler follows Ensembl’s quarterly updates to update all its sources. Since the previous article in 2019 (12), g:Profiler’s current version has doubled the number of supported species and strains to 849 from diverse categories, including vertebrates, plants, insects, fungi and parasites.

### Enrichment analysis using g:GOSt

The default tool in the g:Profiler toolset, g:GOSt, offers an enrichment analysis of gene lists to obtain statistically significant biological functions across various data sources. The data sources include annotations from three GO sub-ontologies—Molecular Function, Biological Process and Cellular Component. This data source is further coupled with Human Phenotype Ontology (4), known annotations from pathway databases of KEGG (17), Reactome (2) and WikiPathways (18), protein expression across tissues from Human Protein Atlas (19), CORUM (3) protein–protein interactions, and predicted regulatory relations from TRANSFAC (20) and miRTarBase (21). For all data sources except for KEGG and TRANSFAC, which are subject to licensing restrictions, users can download all the underlying terms and associations, providing them with greater control over their data and facilitating further analyses.

By default, the query gene lists in g:GOSt are taken as a whole. However, gene lists can be viewed as ordered lists, where the genes are arranged in descending order of significance. The ordered query option is advantageous in cases where genes can be meaningfully ordered based on biological criteria. For example, if performing differential expression analysis, all genes can be ordered by the fold change and used as a g:GOSt input without applying a predefined fold-change threshold. The *P*-value column (*P*_adj_) for ordered queries should not be taken as an indication of statistical relevance but as a score. The primary function of this feature is to determine whether the genes belonging to the term are evenly distributed across the query or predominantly located at the top. This information can be obtained from the ‘Q’ column in the ‘stats’ section of the Detailed results and from the coloured matrix next to the section. In addition, g:Profiler allows for the simultaneous analysis of multiple gene lists using a FASTA-like format. This feature facilitates simple comparisons to determine which biological functions are specific to a particular gene list and which are common.

g:Profiler evaluates the functional enrichment of the input gene list by using the cumulative hypergeometric test, a well-proven method in the field. To mitigate false-positive findings, g:Profiler performs multiple testing correction. The default method is g:SCS (22), but users can also apply the Bonferroni correction or the Benjamini–Hochberg false discovery rate.

Enrichment analysis heavily depends on the background gene list the query is compared against (23). By default, g:GOSt utilizes the set of all annotated protein-coding genes as a background. However, when only a part of the genes are studied due to technological or biological reasons, it would be proper to use a custom background to calculate the statistical enrichment significance. To do this in g:Profiler, users can provide a list of background genes via the ‘Custom’ option from the Statistical domain scope field under the Advanced options of the g:Profiler web page.

**Figure 1.**
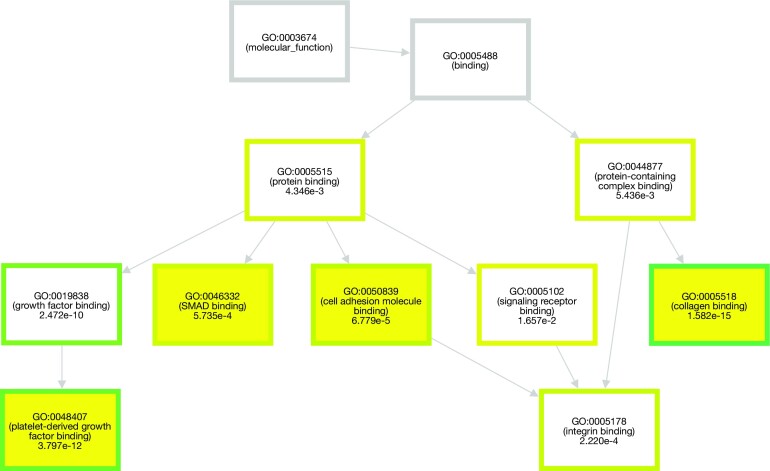
The highlighted driver terms in a broader GO context. The driver terms have a yellow background, other significantly enriched terms have coloured frames corresponding to the enrichment *P*-values, and non-significantly enriched terms providing the broader context and connection to the root term have grey borders. There can be more than one driver term per component. The graph original layout was slightly modified to fit the page.

Upon completing the enrichment analysis in g:GOSt, the resulting enrichment scores are displayed on a Manhattan plot that provides a visual overview of the enriched functional terms and the associated gene lists. This is accompanied by an extensive and interactive table that provides detailed information of the results. The novel GO Context (Figure 1) tab, further described in the New developments section, provides information on the relatedness of the significant terms and their broader context in the GO graph. All these visual outputs can be downloaded in a publication-ready format, providing a convenient way to incorporate the results into scientific publications or presentations.

The g:Profiler web service uses a Python/Flask API backend with a JSON-based API for easy data exchange. SQLite stores data in separate databases for each organism, and roaring bitmaps are used for quick gene set calculations for each term.

### Interoperability of gene lists using g:Convert and g:Orth

The core benefit of relying on Ensembl is the ability to provide seamless identifier mapping for g:GOSt users. This functionality is also available as a separate interoperability service named g:Convert. g:Convert can convert gene identifiers from one namespace to any other among the tens of supported namespaces, such as RefSeq, Illumina, WikiGene and UniProt. The tool supports >100 identifier types for human and at least 30 namespaces for >230 species.

It is evident that not all species are equally well studied, and in many cases, experimental knowledge may be required to translate from more well-studied species to less well-known ones. Ensembl’s mapping of orthologous genes between species becomes a handful in such cases. g:Orth makes this functionality available for g:Profiler users to identify and retrieve orthologous genes between two species. g:Orth provides ortholog mappings only within specific taxonomic groups that are originally provided in separate Ensembl databases, such as Ensembl Fungi and Ensembl Plants.

As with g:GOSt, the input query for both g:Convert and g:Orth can comprise a combination of different identifiers.

### Programmatic access for high-throughput analysis and external tool development

Using a web server is not the most efficient solution for users who generate tens to thousands of gene lists for analysis. Therefore, in addition to our user-friendly web interface, we provide multiple programmatic access points to our services. We provide a JSON-based API over HTTP. In addition, we offer dedicated clients for Python (available via PyPI and conda-forge) and R (available via CRAN and conda-forge), making it easier for users to integrate g:Profiler into their workflows and tools, as proven by >20 external services that use g:Profiler as part of their work. The interactive visualizations familiar from the web server are included in the gprofiler2 R package (24). Furthermore, we have also made g:Profiler available at the Galaxy Tool Shed to support the growing Galaxy community (25).

### Reproducibility

Updates to data can alter analysis results over time, creating potential confusion for colleagues and discrepancies between previously published and new results (11). To address this, all analysis results in g:Profiler are accompanied by metadata that include database versions, selected data sources, query parameters, and the identifiers recognized and used by g:Profiler. These metadata are accessible through the Query Info tab. The information can be saved and shared when publishing the analysis. To simplify sharing and maintain data reproducibility, users can generate a short link that enables anyone with the URL to access query analysis in the same database version in the future. This feature is made possible by the data archives maintained in g:Profiler. The archives go back over 25 Ensembl versions and provide access to data first made available in 2015.

## NEW DEVELOPMENTS IN g:PROFILER IN 2023

### Novel GO term filtering

The increase in produced data and the emergence of powerful new methods have also led to the rapid growth of the size and complexity of knowledge bases, such as the GO. This has contributed to situations where a single gene list can be associated with hundreds of significant functional terms, most of which describe similar processes from different levels of specificity and provide little, if any, additional information. Summarizing and properly interpreting these long lists of terms can be challenging. Therefore, there is a need for methods that help to reduce the amount of information without losing the essential details.

Previously, WebGestalt introduced two redundancy reduction methods in their web tool (6). The weighted set cover determines the smallest gene set subset encompassing all genes from enriched sets, factoring in the *P*-value as the weight or cost of including a set. And the affinity propagation groups gene sets using the Jaccard index for similarity measurement and automatically selects an ‘exemplar’ or representative for each cluster, prioritizing sets with notable *P*-values. However, we find that while these functions might be suitable for small queries where the user expects each gene to be equally crucial, for larger queries trying to find terms that cover the full input list, they might omit smaller and more specific subgroup functions from the ontologies.

In this context, we propose a novel feature of g:Profiler to reduce the redundancy of GO enrichment results. Our proposed two-stage algorithm filters GO enrichment results by first reorganizing significant terms based on their relations into connected components, which are essentially sub-ontologies of GO. This grouping helps to summarize the results, as terms within the same component share similar biological contexts and likely have a large part of the same genes.

This is followed by detecting non-redundant terms from each component using a simple greedy search strategy. This results in identifying the driver gene sets that give rise to other significant functions in the neighbourhood (Figure 1). The greedy search starts from the term with the smallest adjusted *P*-value and excludes child and ancestor terms of the selected term from further searches. The algorithm then recalculates the hypergeometric *P*-value with new parameters, excluding genes already seen in the previous significant term, to determine whether the term remains statistically relevant.

Since the connected components are independent, the greedy search can be applied to them in parallel, resulting in at least one function being presented from every connected component. This approach also ensures that multiple driver terms are considered rather than simply selecting a single term with the highest significance level. Overall, the proposed two-stage algorithm provides a robust and efficient way to filter GO enrichment results.

We evaluated our filtering algorithm by focusing on reducing reported GO terms and preserving biological relevance. Using the GO: Biological Process ontology, we designed queries with varying proportions of related and unrelated genes to assess the algorithm’s performance. Our analysis compared six filtering strategies, including five in-house approaches and one from the SUMER R package (26) with different parameters. The functionality of SUMER is implemented as the weighted set cover feature in the WebGestalt web server (6). It is worth noting that the SUMER strategies require parameters specifying the maximum number of terms to retain, whereas our in-house methods are parameter-free. The strategy currently implemented in g:Profiler outperformed all others, and though SUMER exhibited decent performance, its parameters lacked suitable defaults and did not generalize well across queries. More details about the experiments can be found in Supplementary Data.

### Graph visualizations of GO results

To enhance the interpretability of the relationships between driver terms and the other significantly enriched terms, we have developed a novel graph view as a comprehensive visualization tool for g:Profiler users (Figure 1). This interactive graph view takes advantage of the connected components generated in the previous filtering steps and visualizes each of them individually in a single display. In this view, all significantly enriched terms are shown and connected to the root terms of the three GO domains. A yellow background highlights the driver terms. The non-significant terms, which provide a broader context for the location of terms within the GO graph, are indicated by light grey borders. To accommodate the three domains of GO, we provide three views, allowing easy navigation.

### GMT Helper for custom datasets and species

g:Profiler leverages Ensembl (1) data accessed through the Biomart archive, providing users with accurate and up-to-date information. Although Biomart covers the majority of commonly studied species, the most recent additions to Ensembl for less studied organisms are not always included. Also, there are alternative annotation files that the users might want to use for their analysis, such as those available at Molecular Signatures Database (MSigDb) (27).

To not limit the scope of functional enrichment analyses to the core data sources and species included in g:Profiler, we first introduced the option of uploading custom GMT files in our previous article in 2019 (12).

While MSigDb provides ready-made GMT files, it is not always the case. For example, the GO annotations available via the EMBL-EBI QuickGO website need further modifications to follow the GMT format. To address this issue, we have created a specialized toolkit called GMT Helper (https://biit.cs.ut.ee/gmt-helper).

This toolkit enables users to convert and validate their annotation files for their particular species or data sources. For example, to generate a GMT file that includes annotations from the GO database, users can download annotations from the EMBL-EBI QuickGO website using the corresponding taxon ID. The retrieved data matrix can then be converted into a GMT file using the ‘Convert tabular file to GMT’ function in the GMT Helper. After the GMT file is generated, users can validate its format using the ‘Validate GMT file’ tab and obtain a token for the file that can be used for running queries.

Overall, g:Profiler offers an efficient and effective way to generate custom GMT files for functional enrichment analysis. The custom GMT function has been well received, as demonstrated by being used over 220 000 times in the last 2 years.

## DISCUSSION

In summary, enrichment analysis is a valuable tool in biological research that provides insight into the functions and pathways of a given gene set. However, enrichment analysis has several potential pitfalls that must be considered when interpreting the results (23). One potential issue is the choice of the background set influencing the enrichment results, often unnoticed by the user. A common approach is to use all genes in the organism’s genome as the background set. However, this approach assumes that all genes have the same probability of being selected, which may be incorrect. To overcome this issue, g:Profiler allows to use a custom background set, such as genes expressed in the same tissue or developmental stage as the input gene set.

Another potential pitfall is the redundancy in GO terms. The GO hierarchy is organized in a tree-like structure, where parent terms are more general and child terms are more specific, and all genes related to a child term also belong to the parent terms. This interdependence of the terms leads to long lists of statistically enriched terms from which it is difficult for the researcher to recognize the terms truly driving the full results reported to them. This often results in picking the terms the user expects to see based on their prior personal experience or gut feeling rather than reporting the true signal in the results. To overcome this issue, the novel two-stage algorithm implemented in g:Profiler highlights the true driver terms in a fully automatic manner.

In conclusion, g:Profiler is a widely used and valuable toolset for functional enrichment analysis, conversions between gene identifiers and mappings to their orthologs. With a focus on user-friendly design and comprehensive support for a wide range of evidence types, identifier spaces and organisms, g:Profiler is a reliable and up-to-date resource for researchers in genetics, biology and medical research. The recent update to g:Profiler introduces several novel features, including a two-stage algorithm for filtering GO enrichment results, a graph view for visualizing enriched terms and their relationships within the ontology, and the GMT Helper toolkit for customizing annotations for less commonly studied species. These features make it easier for biologists to obtain a comprehensive summary of their data and understand the biological context of their results. In addition to its web interface, g:Profiler also provides programming interfaces that enable its functionality to be integrated into custom workflows and external tools. It is a valuable resource for researchers who want to develop their own tools or services using g:Profiler. With its powerful features, intuitive interface and focus on user needs, g:Profiler stands out as a leading web service for gene set enrichment analysis.

## DATA AVAILABILITY

g:Profiler is freely accessible at https://biit.cs.ut.ee/gprofiler.

## Supplementary Material

gkad347_Supplemental_File
