# Supplementary material for: g:Profiler—interoperable web service for functional enrichment analysis and gene identifier mapping (2023 update)
Source: Nucleic Acids Res. 2023 May 5;51(W1):W207–12. doi: 10.1093/nar/gkad347 (PMC10320099; doi:10.1093/nar/gkad347)
Supplement: gkad347_Supplemental_File [file gkad347_supplemental_file.pdf]

Supplementary Table 1

Detailed comparison of g:Profiler with other enrichment analysis tools.

Supplementary Table S1. Comparative analysis of functionality in six enrichment analysis tools

	g:Profiler	DAVID	GSEA	Panther.db	WebGestalt	Enrichr
**Enrichment analysis methods**	Cumulative hypergeometric test. A selection of multiple testing correction methods (g:SCS, FDR, Bonferroni)	EASE score (Modified Fisher Exact P-value). A selection of multiple testing correction methods (Bonferroni, Benjamini, and FDR)	GSEA, GSEA-Preranked. A selection of multiple testing correction methods (FDR, NES).	ORA, GSEA. Multiple testing correction (FDR, Bonferroni)	ORA (Over-Representation Analysis), GSEA (Gene Set Enrichment Analysis), NTA (Network Topology-based Analysis)	Fisher's exact test, odds ratio, and a method that combines the two. Benjamini-Hochberg for multiple testing correction
**Supported functional domains**	9	> 40	> 40	4	> 40	> 20
**Number of species supported**	849 species and strains	55464 taxonomies	2 species	143 species	12 species	6 species
**Custom annotations**	Enabled via GMT upload feature and GMT helper tool	No	Yes (GMT)	No	Yes (GMT)	No
**Online/desktop/programmatic interfaces**	Online, and programmatically accessible via API, R package or Python package	Online, programmatically accessible via APIs and web services, R package (external)	Desktop, command line, R package	Online, API	Online, programmatically accessible via API and R package	Online, programmatically accessible via API
**Provides visualisations**	Yes (Manhattan plot and GO connected components)	No	Yes (several options)	Yes (pie charts)	Yes (Table, bar chart, Volcano plot, Enrichment plot)	Yes (Bar graph, Clustergram and Appyter components)
**Interactive**	Yes	No	No	No	Yes	Yes
**Custom background option**	Yes	Yes	No	Yes	Yes	No
**Identifier conversion**	Yes	No	No	No	No	No
**Analysis of multiple queries**	Yes	Yes	No	No	No	No
**Data updates**	Data updated frequently, multiple times per year, since 2007	Frequent updates starting from 2021	Data updated frequently	Data updates are infrequent	Last update: 2019	Inconsistent (updates are introduced as new libraries; latest GO – 2021)
**Usable data archives**	Yes (30, since 2015)	No	No	No	Yes (2017, 2013)	Yes (data versions are listed as data sources, going back to 2013)
**Identifying driver results**	Yes (novel GO term filtering approach)	No	No	No	Yes (weighted set cover and affinity method)	No

This comparison covers information that we were able to find from the publications or the web pages of the listed tools.

Supplementary Materials

Description of the Novel Gene Ontology term filtering.

We propose a novel two-stage hybrid term list filtering algorithm for GO enrichment results that takes into account the underlying topology of annotations, but without introducing additional hyperparameters to tune. The main objective for developing this approach was to systematically reduce the term lists retrieved by g:Profiler.

The first step, once the list of significantly enriched GO functions is detected, is to reorganise the significant terms based on their relations. That is, terms that share GO defined relation, i.e have connecting edges between the term nodes, are grouped together. Therefore, instead of detecting ambiguous clusters of terms by using some, i.e. semantic, similarity measure and a clustering algorithm, we use the reliable knowledge from the manually curated resource of GO. Hereinafter, these term groups are referred to as connected components, and one can think of them as sub-ontologies of GO. The components alone are already helpful for summarizing the results as the terms in the same connected component describe similar biological contexts and most likely share a large part of the genes. These components are shown in the 'GO Context' tab.

The next step is to detect the non-redundant terms from each of these components. We rely on the idea that every component has its leading gene sets that give rise to other significant functions in the neighbourhood. That is, if a child term is significantly enriched, the parent term might appear significant due to the fact that it also includes all the genes from the child term. To detect these leading sets we developed a simple algorithm that greedily starts from the term that has the smallest adjusted p-value and keeps it as a leading term. At the same time, the overlapping genes between the term and the input gene list are remembered as marked genes.

As the GO annotations are propagated up the graph, we now exclude all the child and ancestor terms of the selected term from further searches. We find these by recursively traversing the graph structure of GO. Then the search continues with the next smallest p-value from the remaining functions in the component, but now we implement the idea from the elim method (Alexa et al, 2006) and measure the significance of the term by excluding the marked genes from the term and recalculating the cumulative hypergeometric p-value with the new parameters. With this, we identify whether there are other relevant genes in the query that give rise to the terms representing different biological functions. If this p-value remains significant, that is, it is below the originally set significance threshold, then we keep the term as another leading term and include the overlapping genes to the list of marked genes. The recalculated p-value is used as an indicator in the algorithm and is not reported to the user. Again, we exclude the child and ancestor terms and continue these steps until there are no terms left to check. Since the components are independent and do not share any terms, the greedy search can be applied to the connected components in parallel. As a result, at least one function from every connected component is presented for further interpretation. Though it may seem appealing to simply select only the term with the highest significance level for every component, there might be several leading terms in a component.

Before implementing it for the g:Profiler users, we conducted a straightforward evaluation to assess the performance of our Gene Ontology (GO) filtering algorithm, primarily comparing our in-house developed methods with various parameter settings of the SUMER R package. The primary criteria under examination were: a) the reduction of reported GO terms and b) the preservation of biological relevance within the query. While criterion a) is straightforward to evaluate and quantify, criterion b) is considerably more elusive.

For this investigation, we selected the GO: Biological Process (BP) ontology and designed queries for the experiment. Each query comprised a proportion of genes associated with a random GO:BP term and a proportion of unrelated, random genes. We randomized both the proportions and query size within a reasonable range, allowing us to gauge the impact of "noise" on filtering and evaluate the retention of "signal" in the query both with and without filtering.

Our study analyzed the performance of five in-house developed filtering strategies and the SUMER R package with different parameter settings. The SUMER strategies require parameters specifying the maximum number of terms to

retain, whereas our in-house methods are parameter-free. Ultimately, the strategy currently implemented in the g:Profiler tool outperformed all other contenders, including the SUMER strategies with various parameters. Although the SUMER strategies exhibited commendable performance, their parameters lacked a suitable default and did not generalize well across different queries. (See Supplementary Figure S1).

Furthermore, we conducted the same evaluation using a more complex query featuring signals from two independent GO:BP terms. In this scenario, our implemented filtering strategy demonstrated superior performance, while the limitations of SUMER's parameter-based approach became evident.

A. Alexa, J. Rahnenführer and T. Lengauer, Improved scoring of functional groups from gene expression data by decorrelating GO graph structure, Bioinformatics 22, 1600 (2006)”

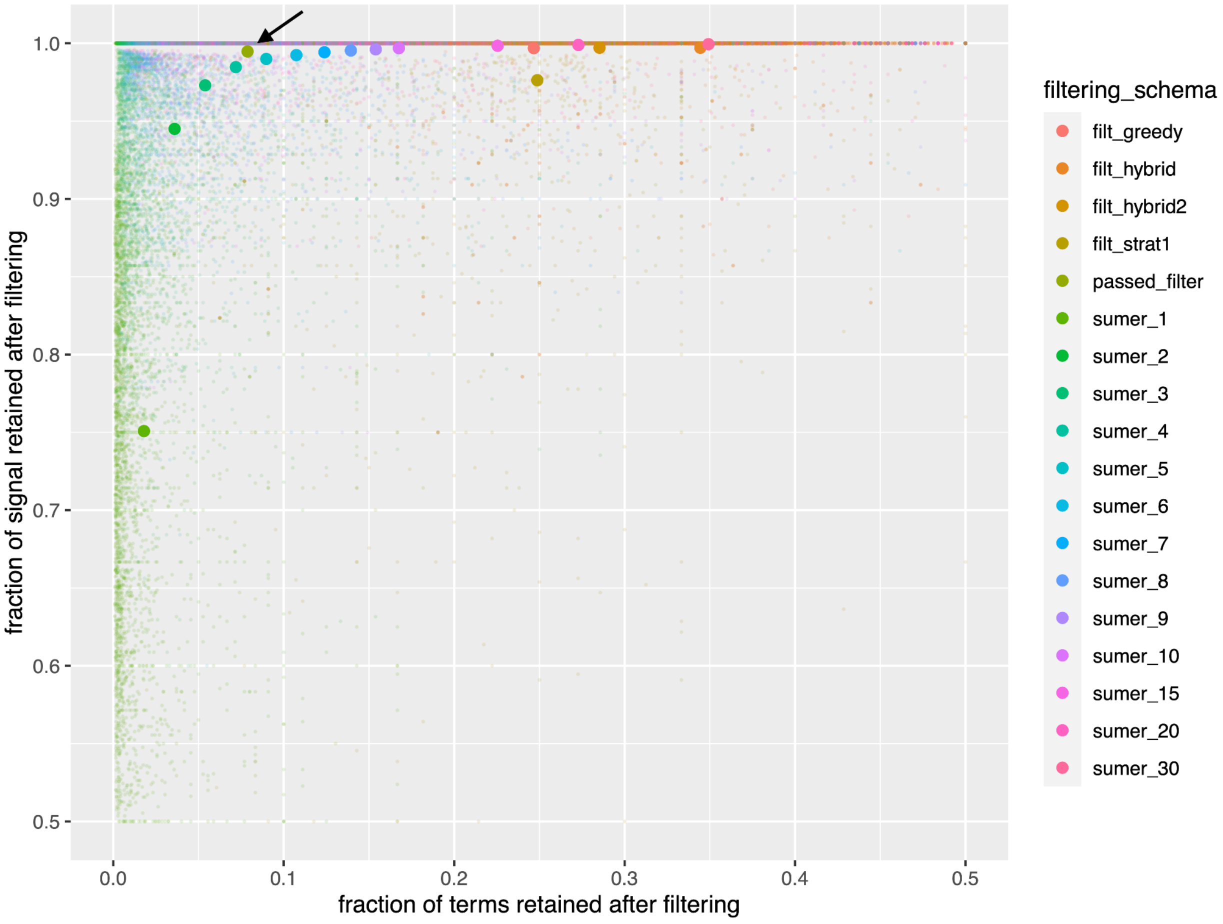

Supplementary Figure S1. Comparison of different filtering strategies on retaining "signal" genes and reducing reported terms in GO queries. The background dot plot indicates all performed queries, while the foreground bigger dots represent the combined average per filtering schema. The x-axis represents the fraction of terms retained after filtering, while the y-axis represents the fraction of "signal" genes with and without filtering. The legend denotes the different filtering methods, including in-house filtering schemes (prefixed with 'filt_'), the currently implemented method in g:Profiler ('passed_filter'), and SUMER methods ('sumer_N', where N denotes the maximum number of terms to retain). The SUMER 'gradient' intuitively visualises the trade-off between signal retention and term reduction. Our 'passed_filter' method, indicated by an arrow, demonstrates the best performance in retaining relevant signal while significantly reducing the number of reported terms.
